# Supplementary material for: The Relationship between Blood Lipids and Risk of Atrial Fibrillation: Univariable and Multivariable Mendelian Randomization Analysis
Source: Nutrients. 2021 Dec 31;14(1):181. doi: 10.3390/nu14010181 (PMC8746968; doi:10.3390/nu14010181)

Supplementary materials

Figure S1 Scatter plot of MR effect size for causal associations between lipid traits and atrial fibrillation

A. HDL-cholesterol and atrial fibrillation

B. LDL-cholesterol and atrial fibrillation

C. Triglycerides and atrial fibrillation

D. Apolipoprotein A1 and atrial fibrillation

E. Apolipoprotein B and atrial fibrillation

Figure S2 Forest plot of MR effect size using MR-Egger and IVW methods for causal associations between lipid traits and atrial fibrillation.

A. HDL-cholesterol and atrial fibrillation

B. LDL-cholesterol and atrial fibrillation

C. Triglycerides and atrial fibrillation

D. Apolipoprotein A1 and atrial fibrillation

E. Apolipoprotein B and atrial fibrillation

Figure S3 Funnel plot of causal associations between lipid traits and atrial fibrillation.

A. HDL-cholesterol and atrial fibrillation

B. LDL-cholesterol and atrial fibrillation

C. Triglycerides and atrial fibrillation

D. Apolipoprotein A1 and atrial fibrillation

E. Apolipoprotein B and atrial fibrillation

Figure S4 Leave-one-out plot to assess if a single variant is driving the association between lipid traits and atrial fibrillation

A. HDL-cholesterol and atrial fibrillation

B. LDL-cholesterol and atrial fibrillation

C. Triglycerides and atrial fibrillation

D. Apolipoprotein A1 and atrial fibrillation

E. Apolipoprotein B and atrial fibrillation

Figure S1 Scatter plot of MR effect size for causal associations between lipid traits and atrial fibrillation

A. HDL-cholesterol and atrial fibrillation

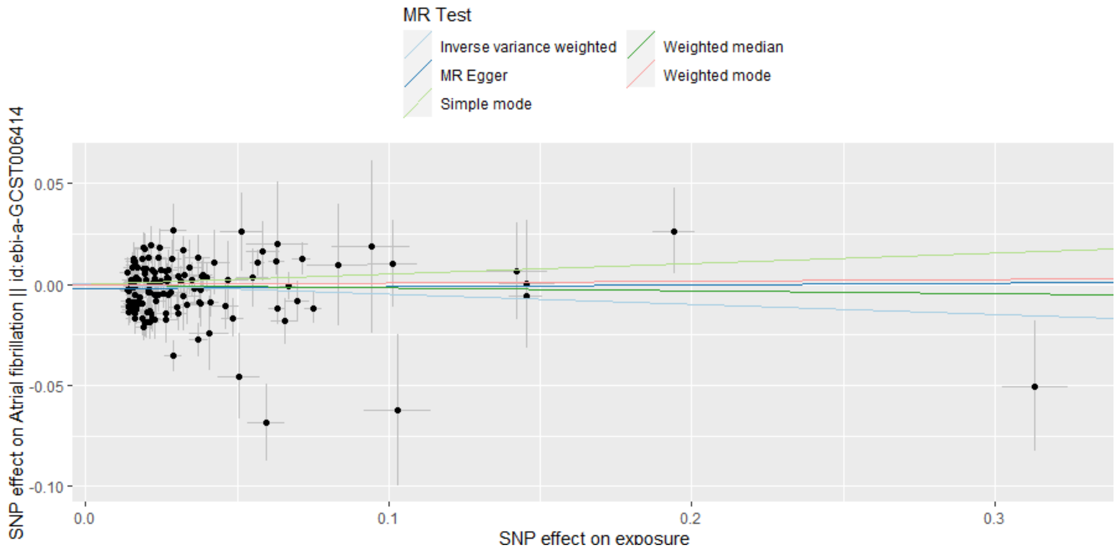

B. LDL-cholesterol and atrial fibrillation

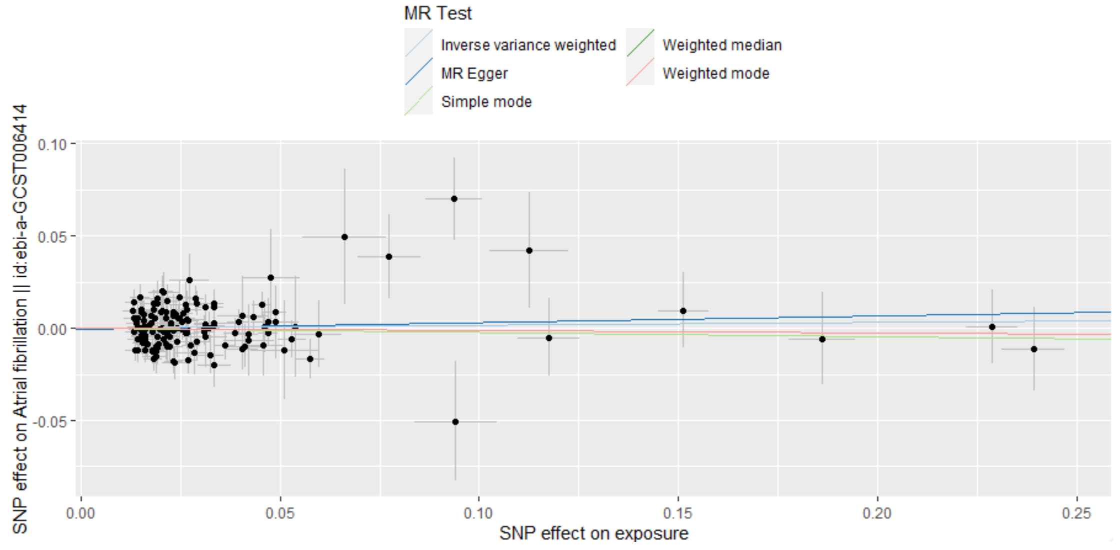

C. Triglycerides and atrial fibrillation

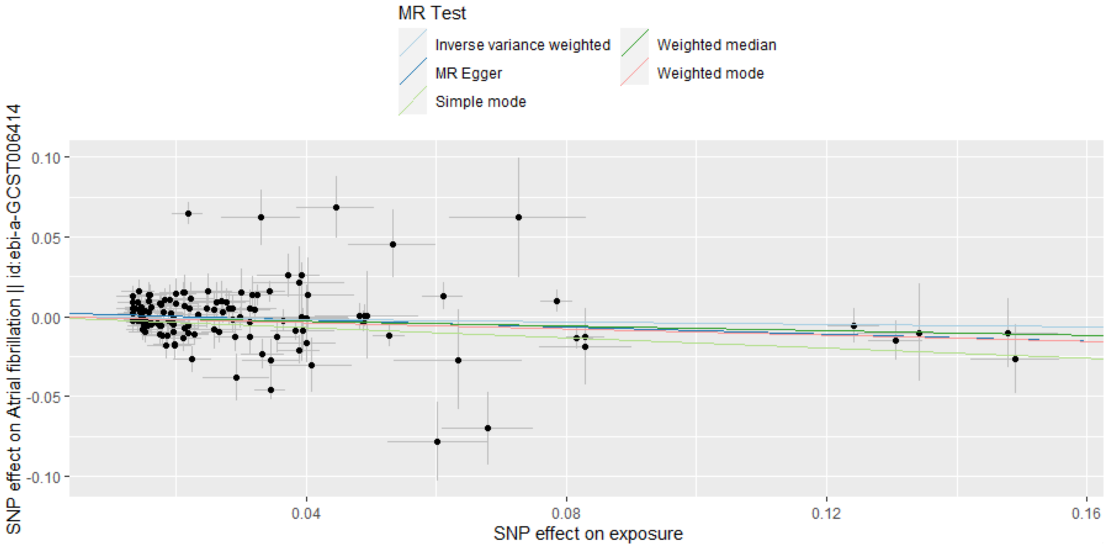

D. Apolipoprotein A1 and atrial fibrillation

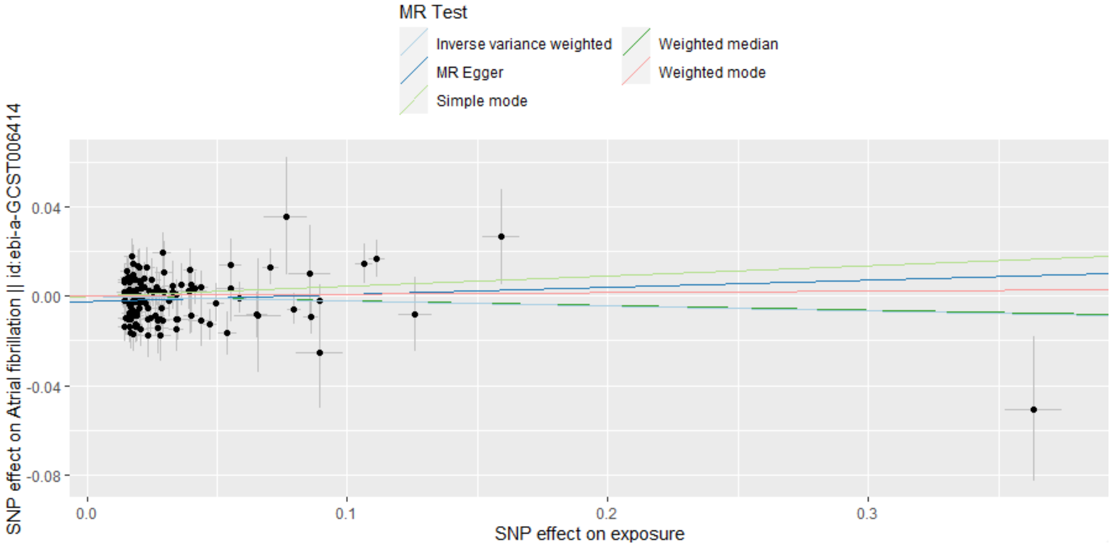

E. Apolipoprotein B and atrial fibrillation

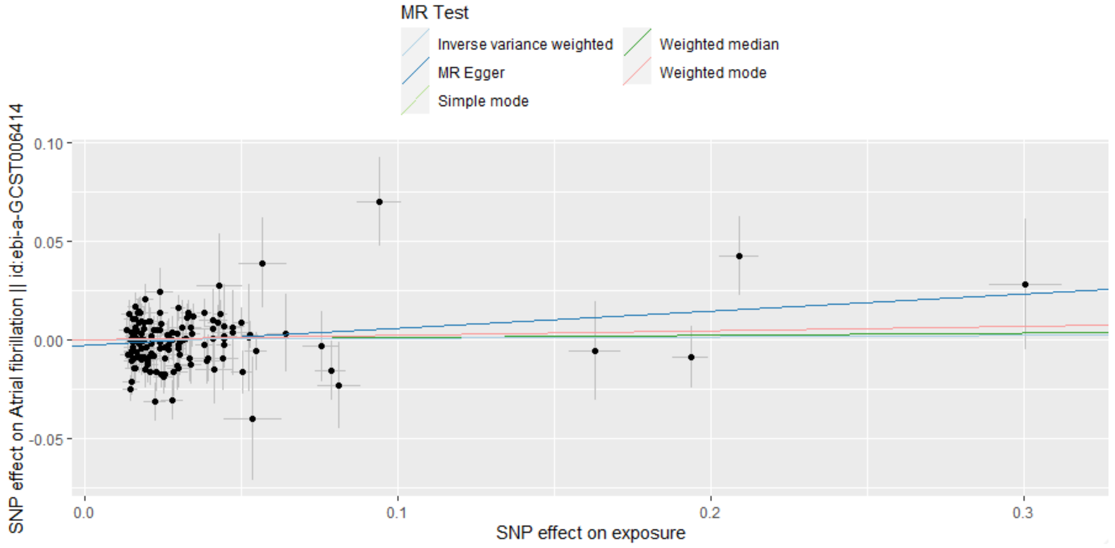

Figure S2 Forest plot of MR effect size using MR-Egger and IVW methods for causal associations between lipid traits and atrial fibrillation.

A. HDL-cholesterol and atrial fibrillation

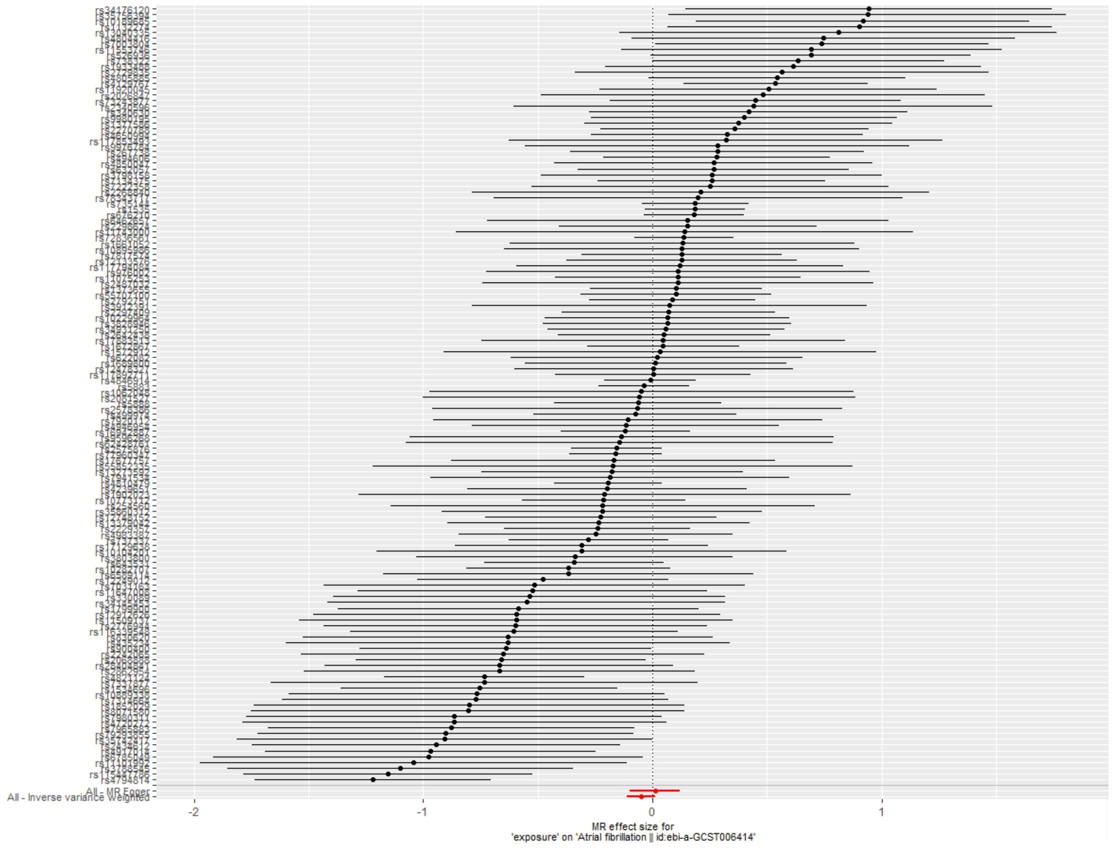

B. LDL-cholesterol and atrial fibrillation

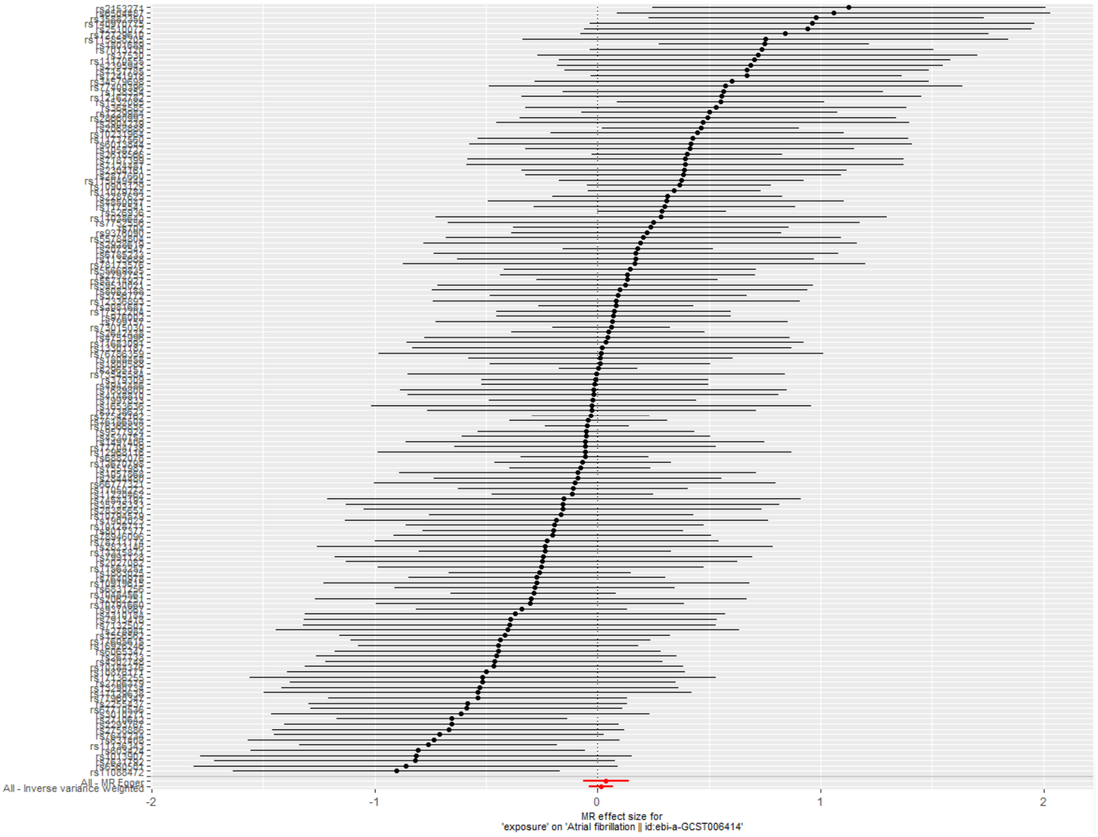

C. Triglycerides and atrial fibrillation

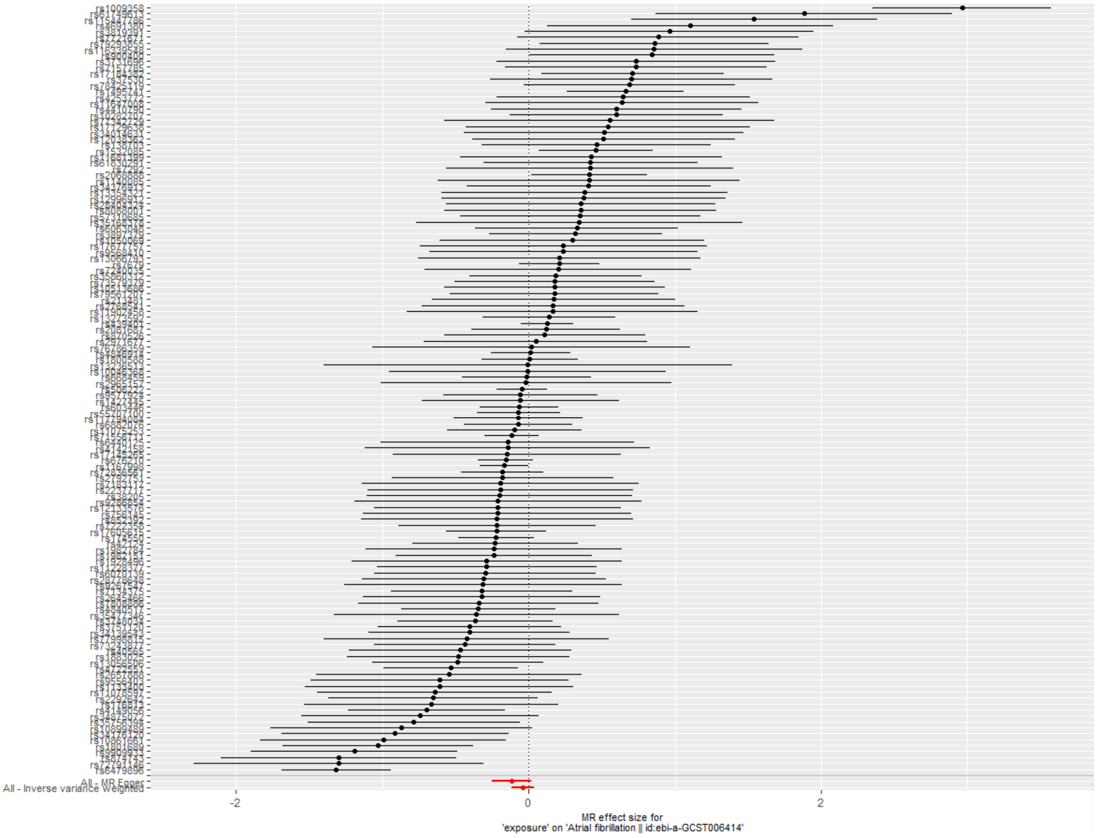

D. Apolipoprotein A1 and atrial fibrillation

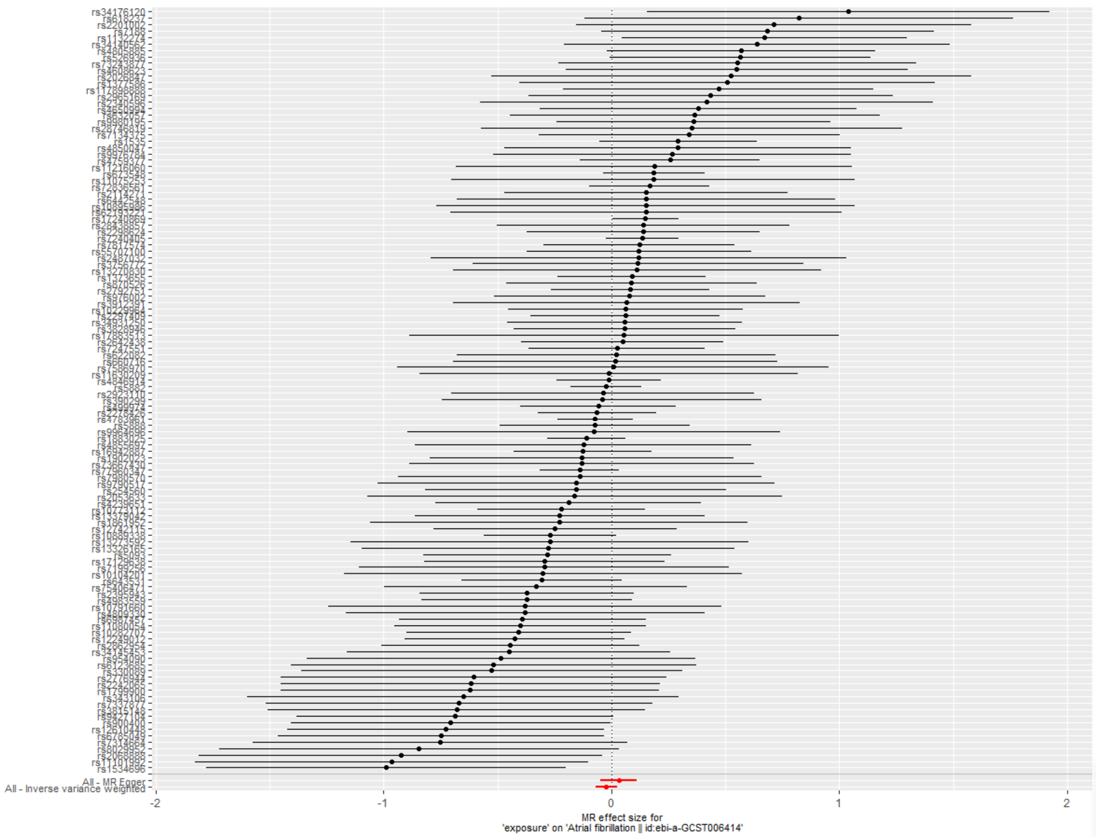

E. Apolipoprotein B and atrial fibrillation

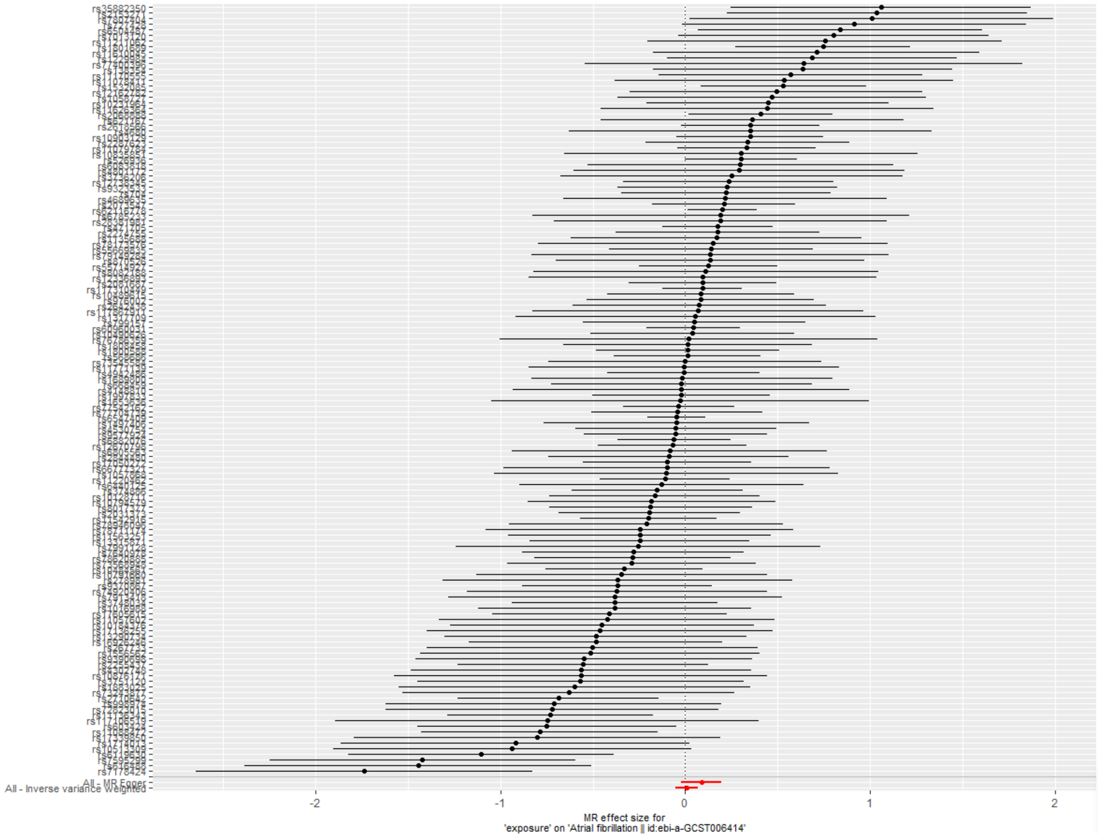

Figure S3 Funnel plot of causal associations between lipid traits and atrial fibrillation.

A. HDL-cholesterol and atrial fibrillation

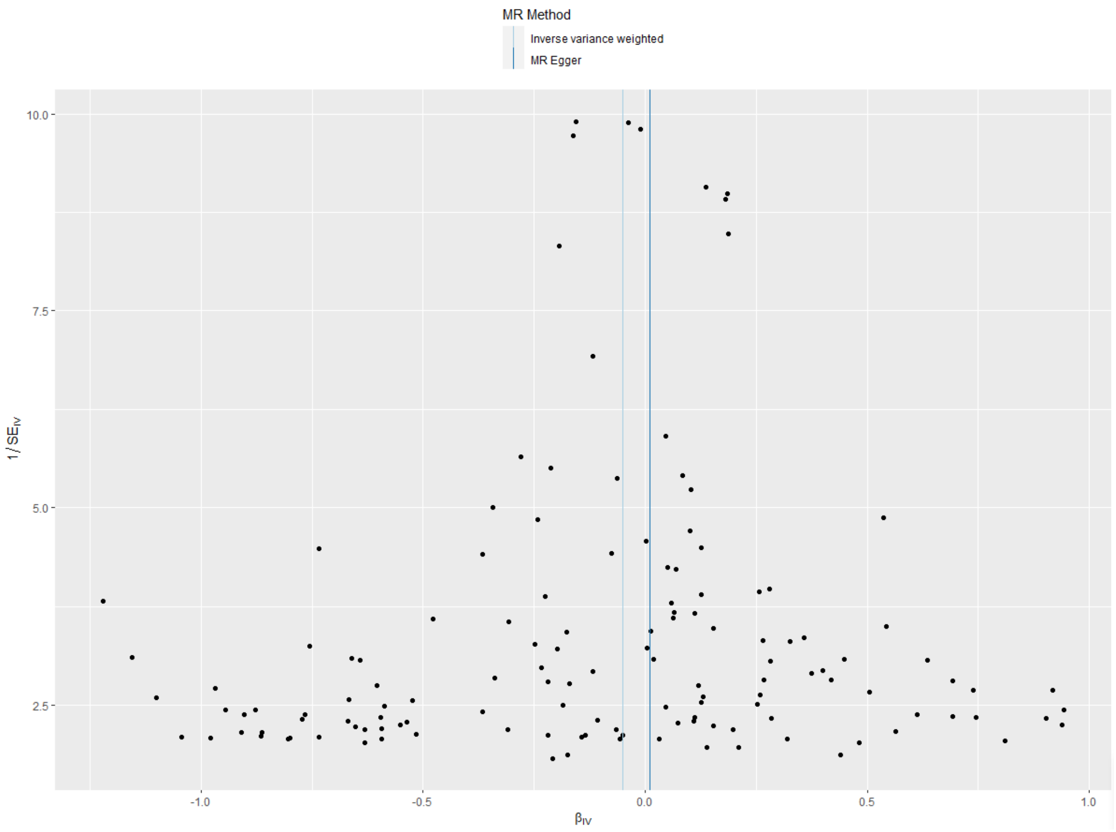

B. LDL-cholesterol and atrial fibrillation

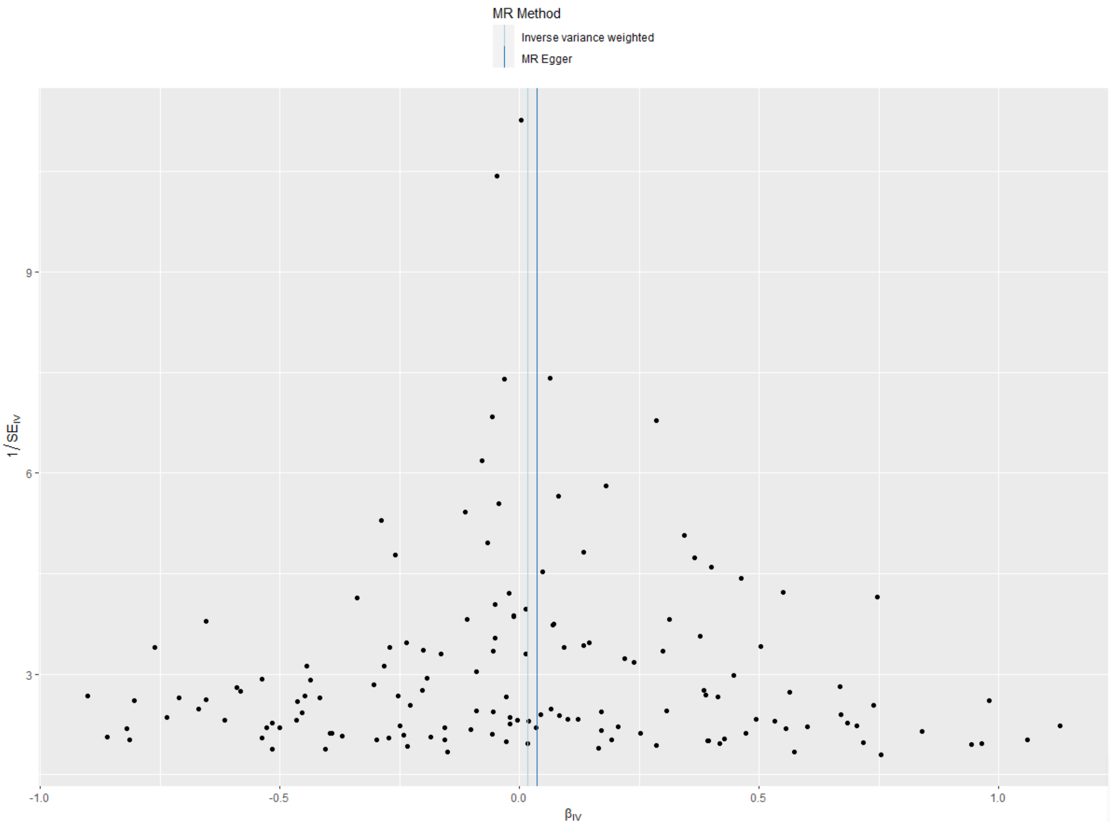

C. Triglycerides and atrial fibrillation

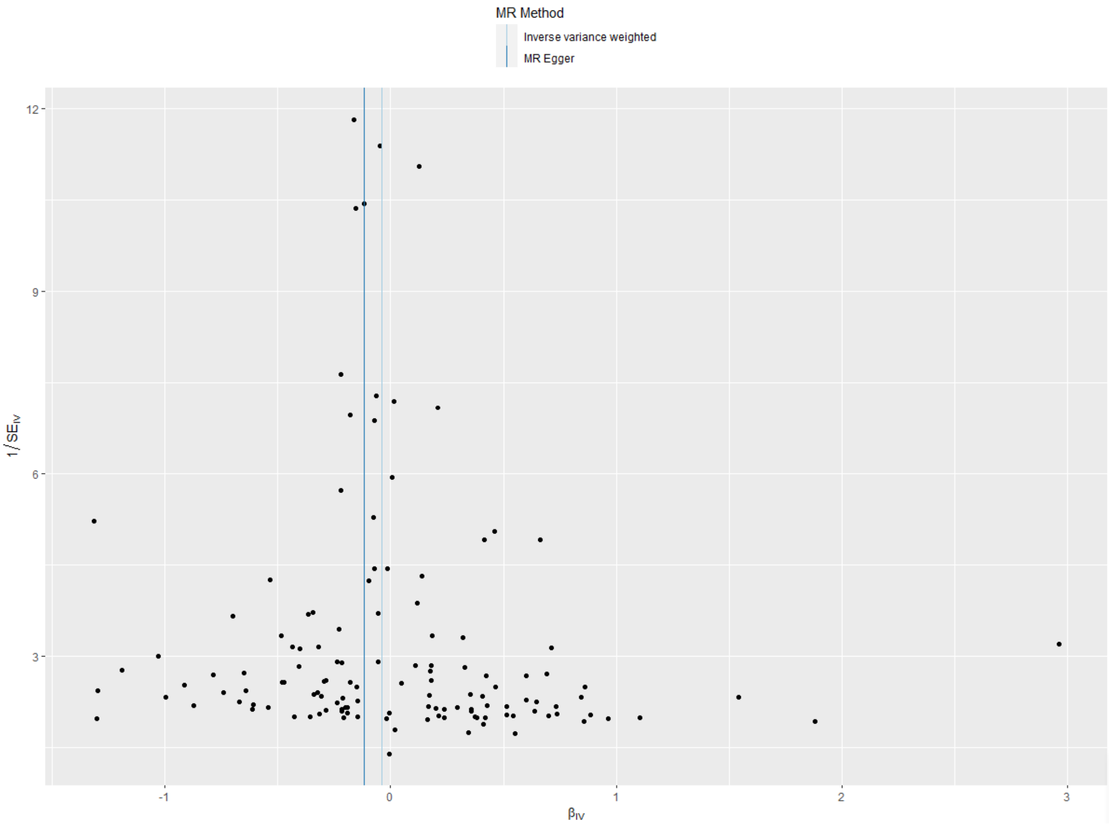

D. Apolipoprotein A1 and atrial fibrillation

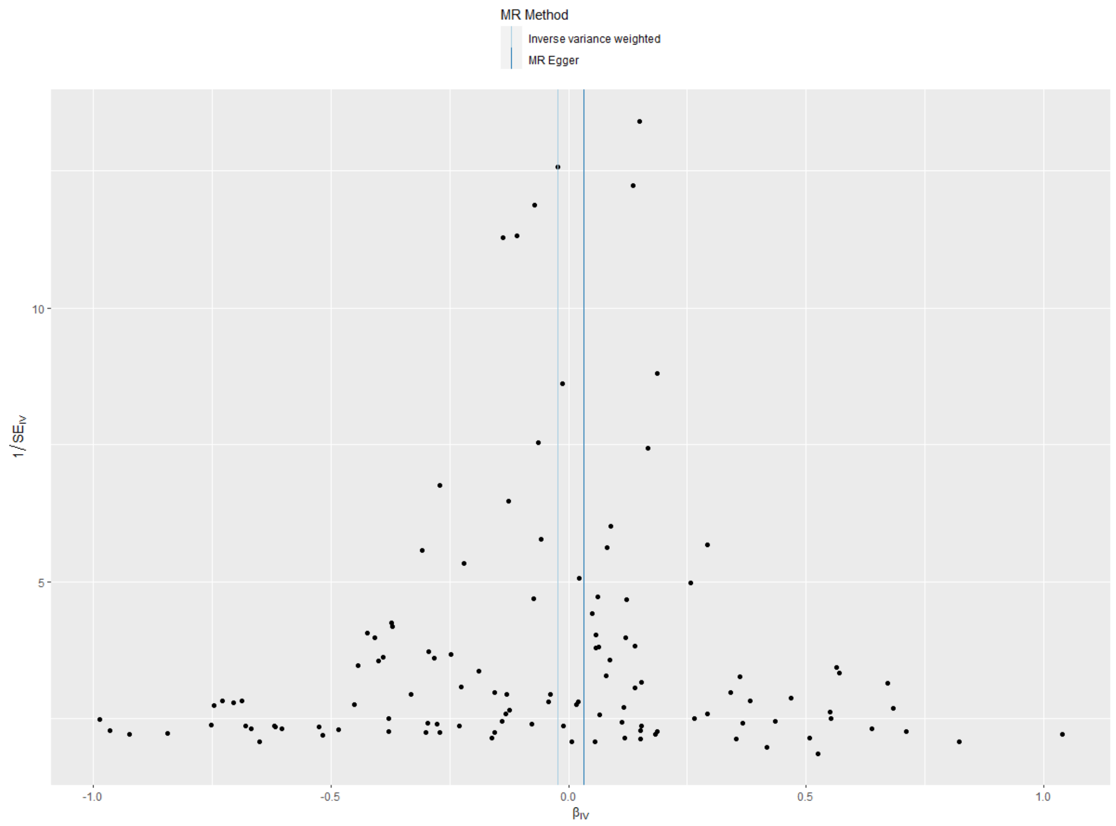

E. Apolipoprotein B and atrial fibrillation

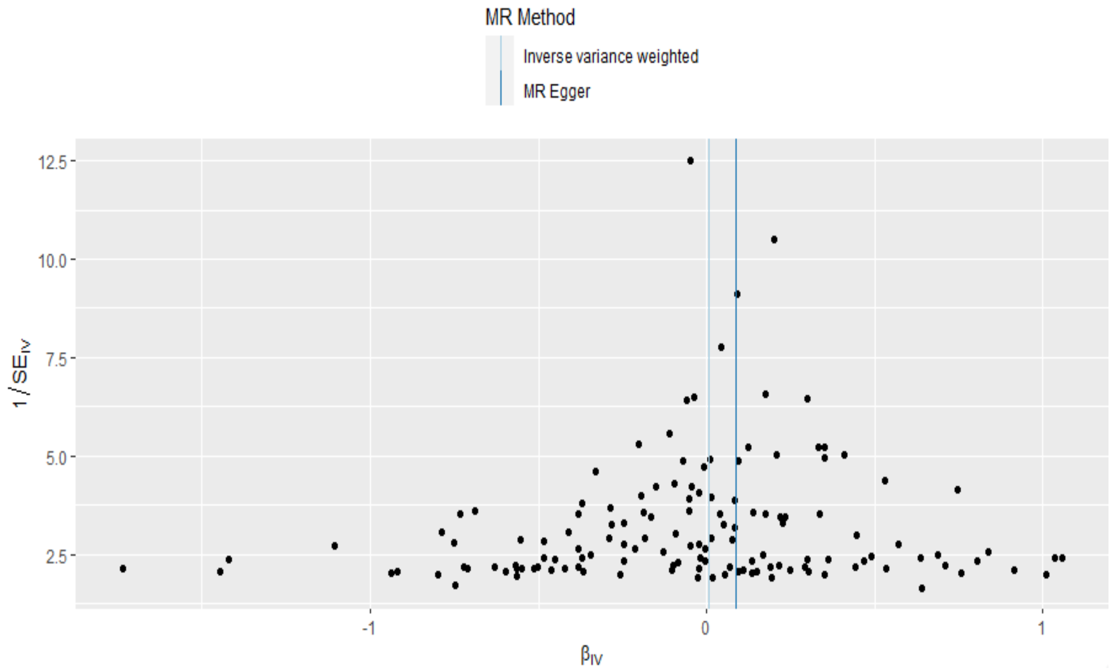

Figure S4 Leave-one-out plot to assess if a single variant is driving the association between lipid traits and atrial fibrillation

A. HDL-cholesterol and atrial fibrillation

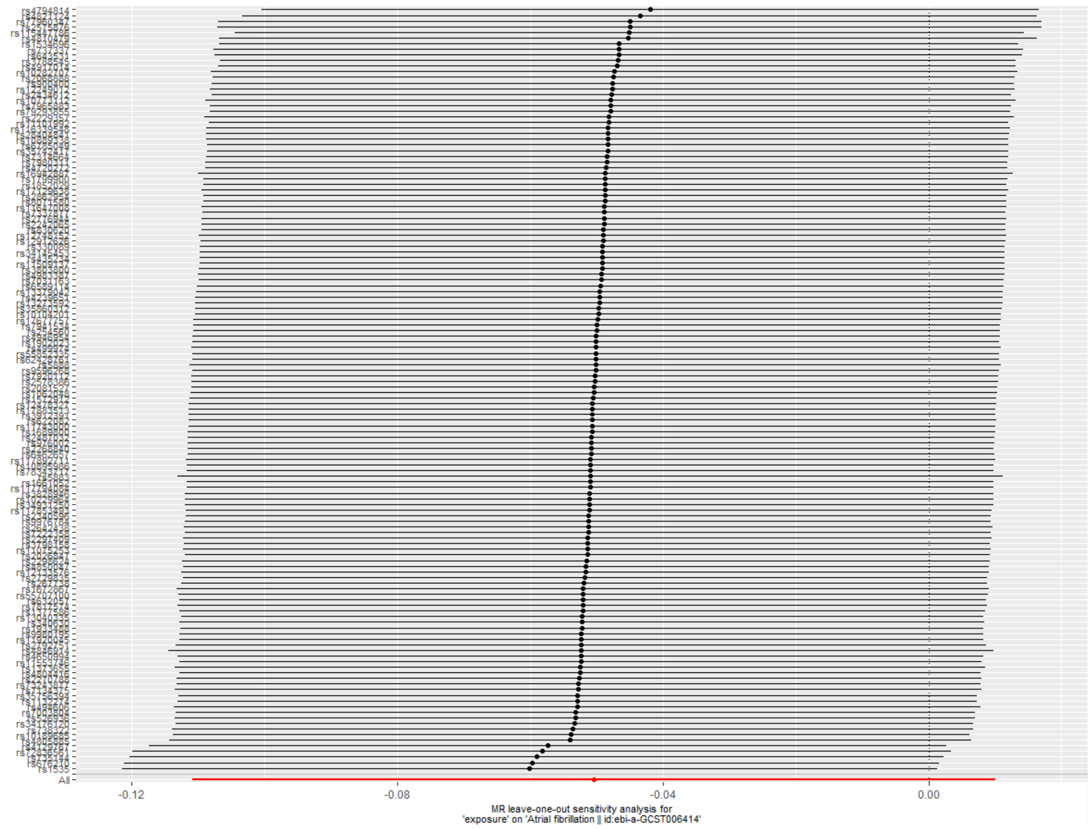

B. LDL-cholesterol and atrial fibrillation

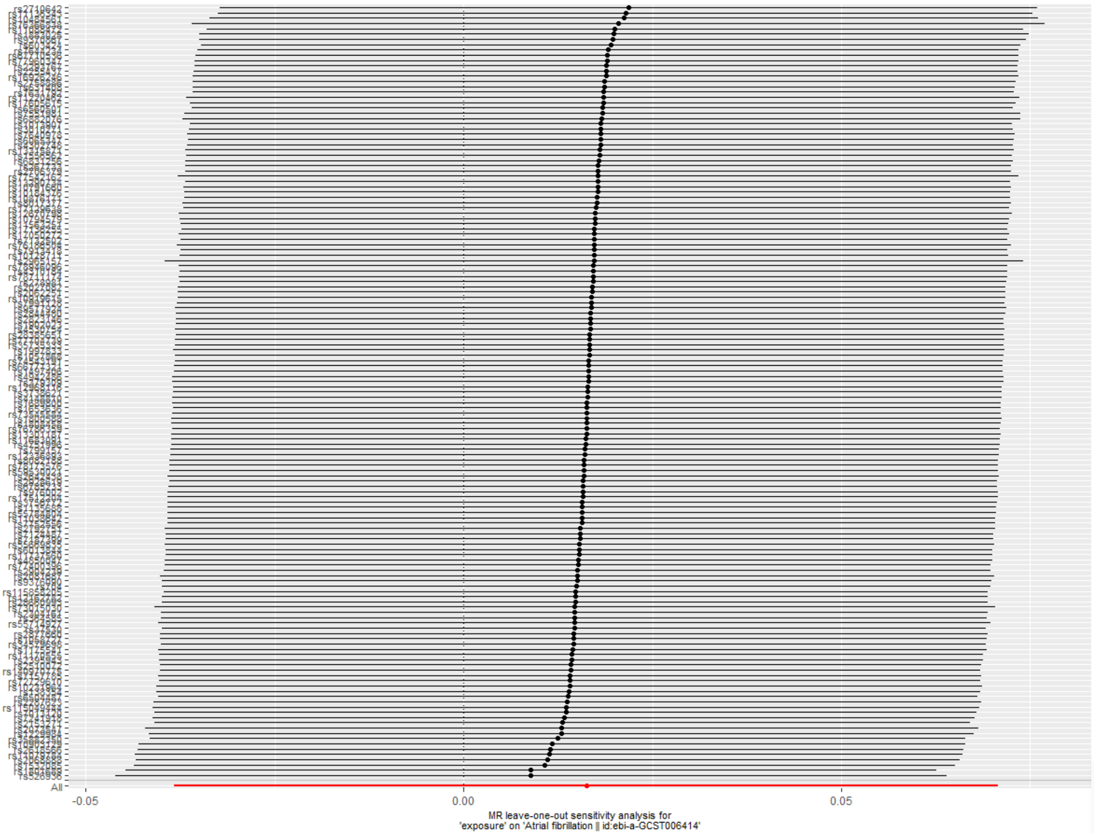

C. Triglycerides and atrial fibrillation

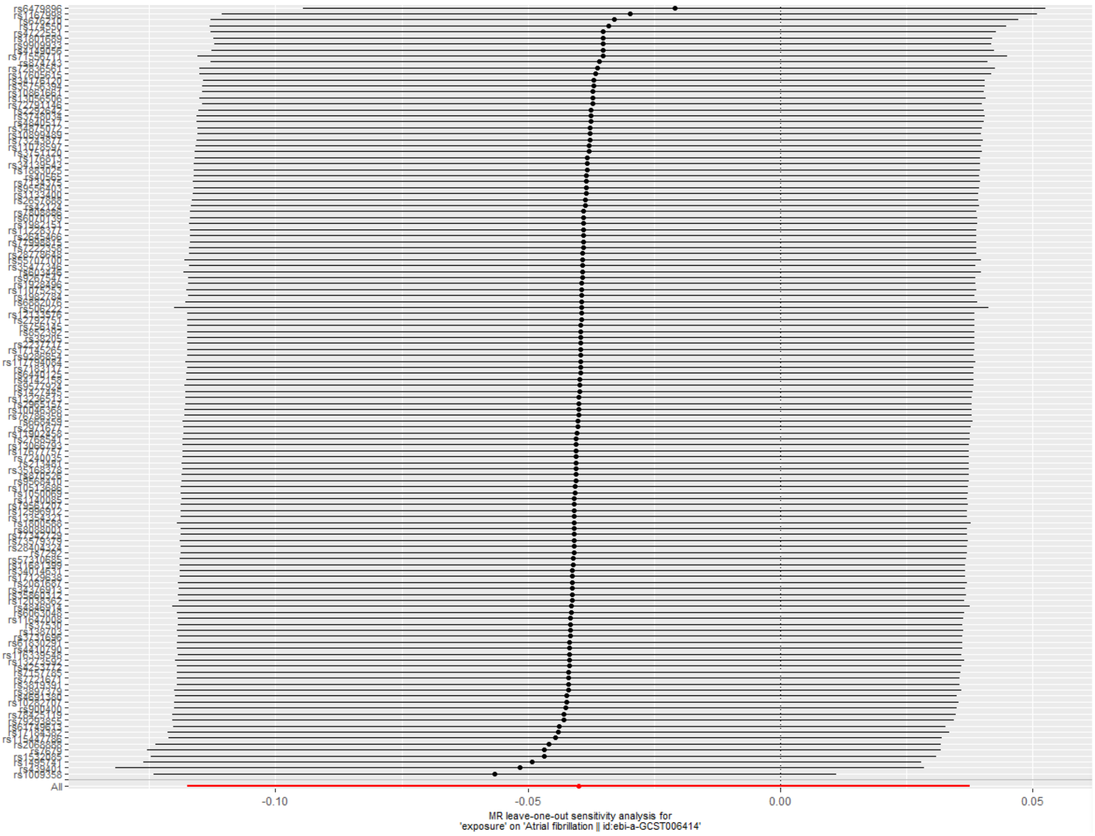

D. Apolipoprotein A1 and atrial fibrillation

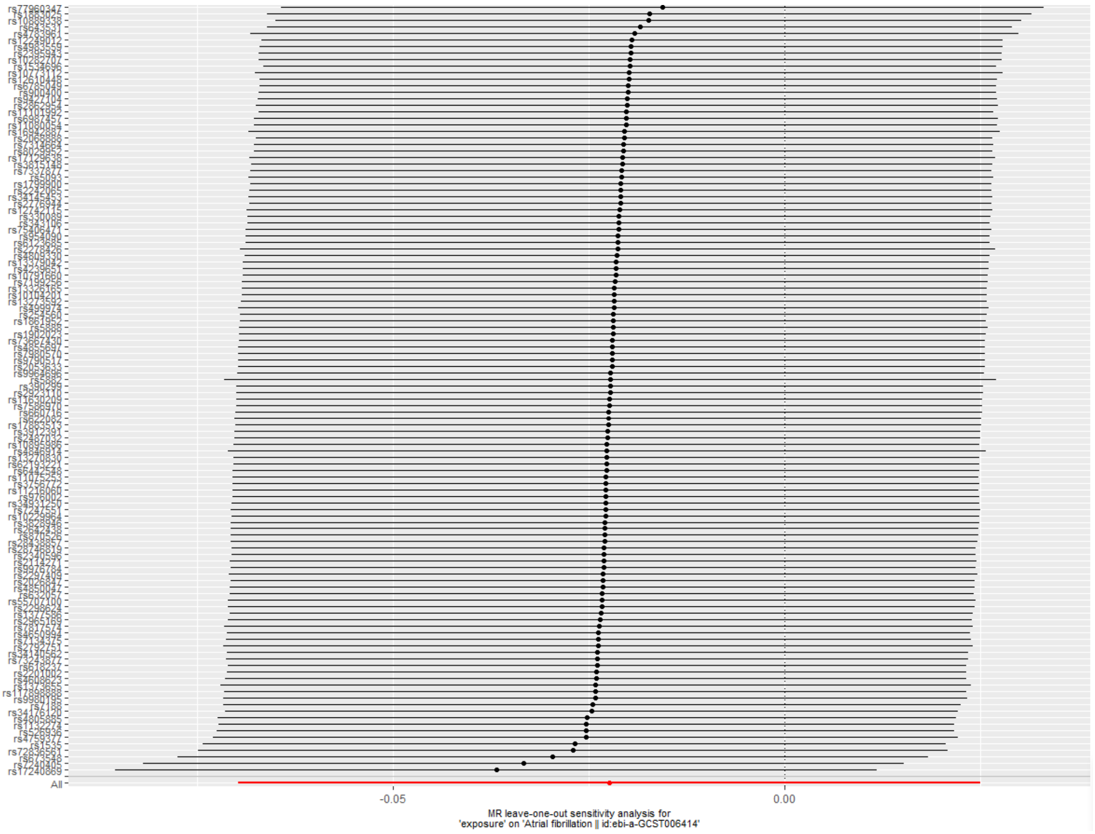

E. Apolipoprotein B and atrial fibrillation

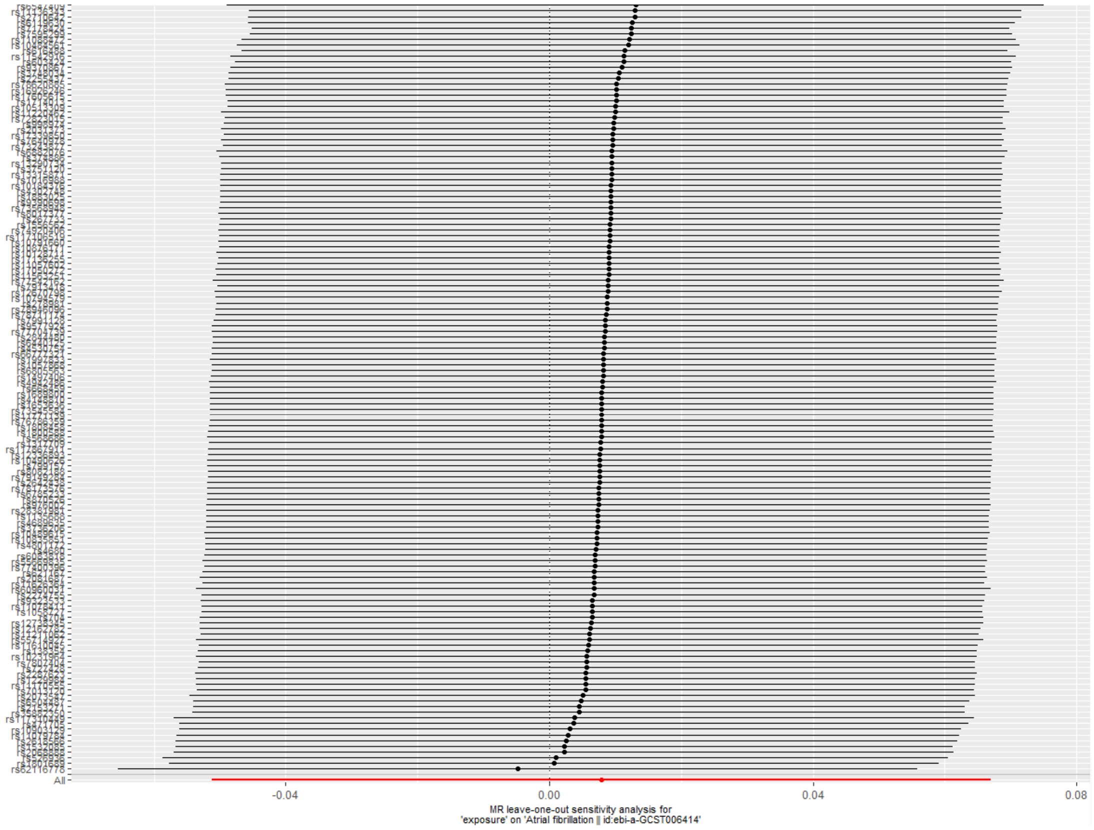

Supplement: Supplementary file 1 [file nutrients-14-00181-s001.zip › Supplementary Figure.pdf]
